# Associations Among Reduced Income, Unhealthy Habits, the Prevalence of Non-Communicable Diseases, and Multimorbidity in Middle-Aged and Older US Adults: A Cross-Sectional Study

**DOI:** 10.3390/healthcare12232398

**Published:** 2024-11-29

**Authors:** Damián Pereira-Payo, Raquel Pastor-Cisneros, María Mendoza-Muñoz, Lucía Carrasco-Marcelo

**Affiliations:** 1 Health, Economy, Motricity and Education (HEME) Research Group, Faculty of Sport Sciences, University of Extremadura, 10003 Cáceres, Spain; 2 Promoting a Healthy Society Research Group (PHeSO), Faculty of Sport Sciences, University of Extremadura, 10003 Cáceres, Spain; 3 Physical and Health Literacy and Health-Related Quality of Life (PHYQoL), Faculty of Sport Science, University of Extremadura, 10003 Caceres, Spain; 4 Department of Financial Economics and Accounting, Faculty of Business, Finance and Tourism, University of Extremadura, Avda. de la Universidad, s/n, 10071 Cáceres, Spain; lucarrascom@unex.es

**Keywords:** epidemiology, economic deprivation, physical activity, public health, socioeconomic inequalities, socioeconomic factors

## Abstract

Introduction: Evidence supports the relationships between socioeconomic status and access to health care, incidence of pathologies, and lifestyle. Objective: The aim of this research was to investigate whether there are associations between having a household income below the poverty line, and participation in unhealthy lifestyle habits, the prevalence of non-communicable diseases, and the number of comorbidities in US middle-aged and older adults. Methods: This cross-sectional study is based on the NHANES 2011–2020. A total of 10,788 US middle-aged and older adults (5653 males and 5135 females) participated in this research. Associations were studied through the Chi-squared test, and odds ratios were calculated using a binary logistic regression model. Results: There were associations between a household income below the poverty line and physical inactivity, unhealthy diet, and being or having been an alcoholic. Associations were found between this adverse economic situation and having hypertension, diabetes, liver disease, kidney problems, arthritis, congestive heart failure, angina pectoris, heart attack, stroke, and also with having two or more, three or more, four or more, and five or more comorbidities. Increased odds of being involved in these unhealthy habits and of suffering these diseases and multimorbidity were found for those with a family income below the poverty threshold. Conclusions: The existence of associations between having a family income under the poverty threshold and having unhealthy habits, suffering non-communicable diseases, and having multimorbidity is confirmed in US middle-aged and older adults. Increased odds for various non-communicable diseases, multimorbidity, and for being involved in these unhealthy habits were found for this low-income group. These findings should serve to draw the attention of policy makers to the increased health vulnerability of the adult population below the poverty line in the US.

## 1. Introduction

Socioeconomic status (SES) describes the situation of individuals based on their education, income, and type of work, usually categorized as high, medium, or low. In other words, socioeconomic status could be defined as a person’s social place within a social group based on factors such as income or education [1].

In many cases the accessibility that individuals may have to certain services is determined by their socioeconomic level or status; for example, in the case of housing [2], health services such as health insurance [3], and access to certain levels of the education system [4]. This condition of inability, in which an individual may have limited access to societal resources, is known as socioeconomic deprivation, and is directly linked to factors of poverty, discrimination, or other disadvantages [5,6]. This socioeconomic deprivation also affects access to the health system, with socioeconomic inequalities also having an impact on the health of individuals.

There are different methods of health care management that each country administers and finances. Most health systems include a combination of public and private health care providers [7]. In the case of the United States, it has a combination of public and private health services, with the private part predominating. Thus, the main weakness of the system is the insufficiency of insurance coverage for high-cost health problems, as there is a proportion of Americans who are uninsured or underinsured due to their incapacity to assume the cost of medical insurance services [8].

Although total health expenditure per capita in the United States is one of the highest among members of the Organisation for Economic Co-operation and Development, life expectancy is lower than in member countries. In addition, there is racial and ethnic disparity in mortality rates [9].

This highlights the difficulty of a certain part of the population to access certain care services due to factors linked to their economic and social status. Numerous studies show higher mortality and prevalence of chronic diseases such as diabetes [10], stroke [11], hypertension [12] as well as cholesterol [13], thyroid [14] and liver disease [15] in individuals at risk of socioeconomic exclusion Also, a direct impact is observed among people of low socioeconomic status linked to unfavorable mental health status [16]. Furthermore, it has been observed that people with unfavorable economic status suffer from a higher number of comorbidities [17,18].

On the other hand, lifestyle habits have been demonstrated to have implications for health and quality of life in the short and long term [19,20]. An individual’s lifestyle is partially determined by their environmental and socioeconomic context, even more during adolescence and the early phases of adulthood [21]. In line with this, economic conditions, especially poverty, are considered relevant factors in the consolidation of health patterns that will be maintain over time [22]. Physical inactivity [23], an unhealthy diet [24], and alcohol consumption [25] are among the modifiable lifestyle factors that can impact health in a more negative way.

Evidence suggests the existence of a relationship between an individual’s socioeconomic level and their ability to access health care, their lifestyle, and their incidence of pathologies. Although there is evidence of an association between each of these variables separately and socioeconomic status, there has no study of the US population that includes the associations between household income and behavioral factors related to healthy habits, the prevalence of noncommunicable diseases, and comorbidities, and that calculates odds ratios for these variables.

We could hypothesize that those people with an income below the poverty line could have a higher prevalence of diseases and a higher participation in unhealthy lifestyle habits. Thus, the aim of this study was to study, in a population of US middle-aged and older adults, whether there are associations between having a family income below the poverty threshold and participation in unhealthy lifestyle habits, the prevalence of non-communicable diseases and multimorbidity. In addition, the odds ratios of having unhealthy lifestyle habits, suffering from non-communicable diseases, and having several comorbidities were calculated.

## 2. Materials and Methods

### 2.1. Design

The present work is a cross-sectional study that uses data from the National Health and Nutrition Examination Survey (NHANES), from its versions since 2011 to March 2020. The NHANES is a program conducted by the National Center for Health Statistics (NCHS), whose objective is to address the nutritional and health status of non-institutionalized US adults and children [26].

### 2.2. Participants

The following inclusion criteria were established for participants to enter the final sample: being 40 years old or older and having valid data for the variables: ratio of family income to poverty, physical activity, quality of diet, alcohol consumption, hypertension, high cholesterol, diabetes, liver conditions, thyroid problems, kidney problems, arthritis, congestive heart failure, coronary heart disease, angina pectoris, heart attack, and stroke.

The NHANES, in its editions from 2011 to March 2020, has a total of 45,462 participants (22,472 males and 22,990 females). In total, 27,845 participants were excluded because they were younger than 40 years old. A total of 6829 participants were excluded due to the unavailability of data on one or more of the following variables: ratio of family income to poverty, physical activity, diet, alcohol consumption, hypertension, cholesterol, diabetes, congestive heart failure, coronary heart disease, angina pectoris, heart attack, stroke, thyroid problems, liver condition, kidney problems, and arthritis.

**Figure 1:**
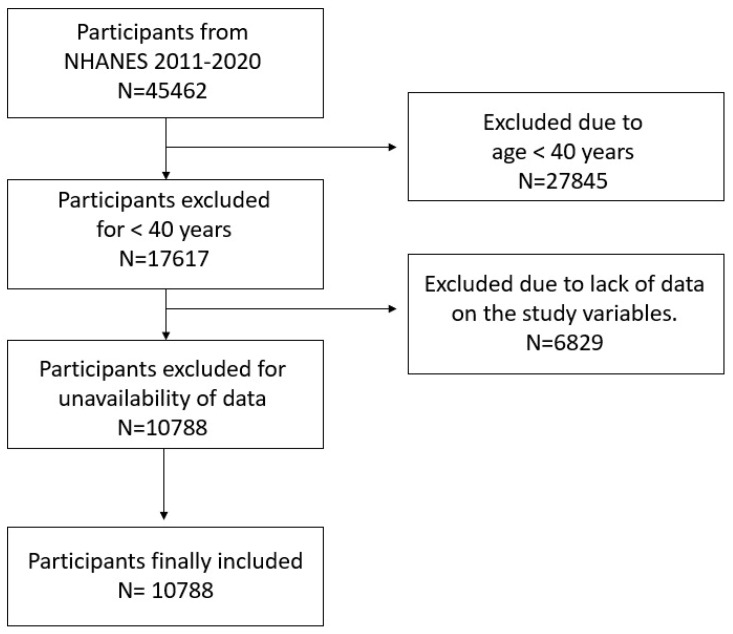
Flow diagram.

The final sample consisted of 10,788 participants (5653 males and 5135 females) (Figure 1).

### 2.3. Variables

- Gender (item RIAGENDR of the NHANES): participants selected one option among: “males” and “females”.
- Age (item RIDAGEYR of the NHANES): in years at the moment the survey and a medical examination were conducted.
- Family income (item INDFMPIR of the NHANES): This variable was generated from the ratio of family income to poverty variable (item INDFMPIR), which expresses a family’s income as a function of the poverty threshold in its state. The poverty threshold corresponds to 1. All families with a value below 1 are below the poverty threshold.
- Physical Inactivity: This variable sought to classify the participants as “physically inactive”, in the case of those who reported not practicing any type of physical activity, or “physically active”, for those who reported walking, cycling or engaging in moderate/intense physical activity. This variable was constructed from the Generalized Physical Activity Questionnaire [26] (items PAQ610, PAD615, PAQ625, PAD630, PAQ640, PAD645, PAQ655, PAD660, PAQ670, and PAD675 of the NHANES).
- Unhealthy diet: Participants were asked to define their diet as “Excellent”, “Very Good”, “Good”, “Fair”, or “Poor”. Participants who answered with one of the three first options (“Good”, “Very Good”, or “Excellent”), were classified as having a “healthy diet”, while individuals who responded “Fair” or “Poor”, were identified as having an “unhealthy diet”. This variable was generated from the item DBQ700.
- Alcohol drinker: Participants were asked if they experienced a time in their life when they drank four or more drinks of any alcoholic beverage almost daily. They had to answer “Yes” or “No”. This variable corresponds to item ALQ151.
- Hypertension: “Yes” or “No” depending on whether participants had been diagnosed with hypertension by a physician. This variable corresponds to item BPQ020.
- High cholesterol: “Yes” or “No” depending on whether participants had been diagnosed with high cholesterol by a physician. This variable corresponds to item BPQ080.
- Diabetes: “Yes” or “No” depending on whether participants had been diagnosed with diabetes by a physician. This variable corresponds to item DIQ010.
- Liver condition: “Yes” or “No” depending on whether participants had been diagnosed with a liver condition by a physician. This variable corresponds to item MCQ160L.
- Thyroid problems: “Yes” or “No” depending on whether participants had been diagnosed with thyroid problems by a physician. This variable corresponds to item MCQ160M.
- Kidney problems: “Yes” or “No” depending on whether participants had been diagnosed with kidney problems by a physician. This variable corresponds to item KIQ022.
- Arthritis: “Yes” or “No” depending on whether participants had been diagnosed with arthritis by a physician. This variable corresponds to item MCQ160A.
- Congestive heart failure: “Yes” or “No” depending on whether participants had been diagnosed with congestive heart failure by a physician. This variable corresponds to item MCQ160B.
- Coronary heart disease: “Yes” or “No” depending on whether participants had been told by a physician that they had coronary heart disease. This variable corresponds to item MCQ160C.
- Angina pectoris: “Yes” or “No” depending on whether participants had been told by a physician that they had angina pectoris. This variable corresponds to item MCQ160D.
- Heart attack: “Yes” or “No” depending on whether participants had been told by a physician that they had heart attack. This variable corresponds to item MCQ160E.
- Stroke: “Yes” or “No” depending on whether participants had been told by a physician that they had coronary heart disease. This variable corresponds to item MCQ160F.
- Number of comorbidities: For the calculation of this variable, the participants’ response to the variables hypertension, hypercholesterolemia diabetes, liver condition, thyroid problems, kidney problems, arthritis, congestive heart failure, coronary heart disease, angina pectoris, heart attack, and stroke, was taken into account. One point was added for each pathology suffered by the participant, the total being the number of pathologies suffered by the subject. The following groups were formed—the grouping of the participants by the number of comorbidities they suffered from:

No morbidities: the participant did not suffer from any of the pathologies included in this research.

One or more comorbidities: the participant suffered from at least 1 of the pathologies included in this study.

Two or more comorbidities: the participant suffered from at least 2 of the pathologies included in this study.

Three or more comorbidities: the participant suffered from at least 3 of the pathologies included in this study.

Four or more comorbidities: the participant suffered from at least 4 of the pathologies included in this study.

Five or more comorbidities: the participant suffered from at least 5 of the pathologies included in this study.

### 2.4. Statistical Analysis

Data normality was tested using the Shapiro–Wilk test (*p* < 0.001) and Q-Q representation. Sufficient evidence was not found to assume that data followed a normal distribution, this suggested that non-parametric tests should be used in the next statistical procedures.

The ordinal variables are presented as number of cases and percentage. Age, which is the only continuous variable, was presented in the median and interquartile range (and mean and standard deviation as complementary data).

Associations were studied through the Chi-squared test, exploring the possible associations between an income under the poverty threshold and non-communicable diseases, non-healthy habits, and suffering comorbidities. To study differences in proportions between those with a family income under the poverty threshold and those above it, the post hoc pairwise z-test for independent proportions was used. Additionally, in order to study the effect size, Phi was calculated.

The odds ratios and their 95% confidence intervals adjusted for age and gender, were calculated using the generalized linear models with binomial distribution with the backward LR method.

The level of significance was set at 0.05, and for all the mentioned statistical procedures, the software SPSS 26th version (IBM SPSS, Chicago, IL, USA) was used.

## 3. Results

In Table 1, the mean and median age of the participants, and the number of participants with a family income above/equal or below the poverty threshold can be observed. Females were shown to be significantly younger than males (<0.001). No significant differences regarding family income were found among genders (*p* = 0.496).

**Figure 2:**
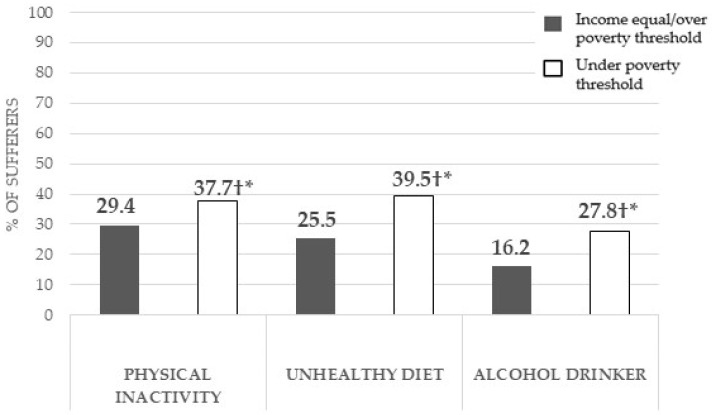
Prevalence of unhealthy habits in middle-aged and older adults with a family income under the poverty threshold, compared to those with a higher income, and associations of having these unhealthy habits with an income under the poverty threshold. † = Significant associations according to the Chi-squared test between having an income below the poverty threshold and the prevalence of a non-communicable diseases; * = significant differences in the z-test with Bonferroni corrections between participants with an income below the poverty threshold compared to those equal to or above it.

The Chi-squared test showed that there were significant associations between having a family income below the poverty threshold and not being physically active (<0.001), having an unhealthy diet (<0.001) and being or having been a high alcohol consumer (<0.001) (Figure 2). Likewise, participation in unhealthy lifestyle habits was significantly higher in people with an income below the poverty line, with this group having a higher percentage of physically inactive people, with an unhealthy diet, and who drink or have drunk large amounts of alcohol, compared to those with an income equal to or above the poverty line.

**Figure 3:**
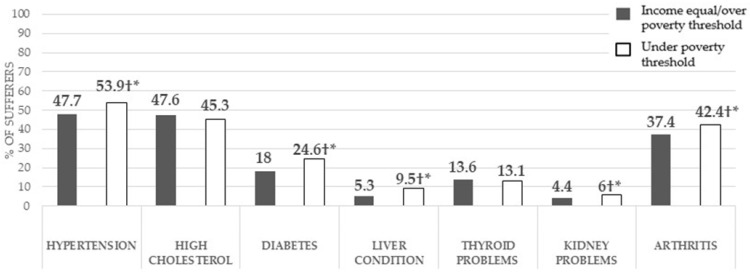
Prevalence of non-communicable diseases in middle-aged and older adults with a family income under the poverty threshold, compared to those with a higher income, and associations of these diseases with an income under the poverty threshold. † = Significant associations according to the Chi-squared test between having an income below the poverty threshold and the prevalence of a non-communicable disease; * = significant differences in the z-test with Bonferroni corrections in the prevalence of a non-communicable disease between participants with an income below the poverty threshold compared to those equal to or above it.

Similarly, an income below the poverty line was proven to be associated with the prevalence of hypertension (<0.001), diabetes (<0.001), liver problems (<0.001), kidney problems (<0.05), and arthritis (<0.001) (Figure 3). Individuals with a family income below the poverty line had a significantly higher prevalence of hypertension, diabetes, liver problems, kidney problems, and arthritis.

**Figure 4:**
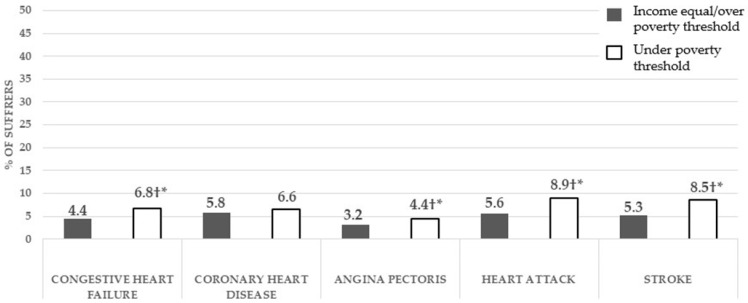
Prevalence of cardiac diseases in middle-aged and older adults with a family income under the poverty threshold, compared to those with a higher income, and associations of this diseases with an income under the poverty threshold. † = Significant associations according to the Chi-squared test between having an income below the poverty threshold and the prevalence of a cardiac problem; * = significant differences in the z-test with Bonferroni corrections in the prevalence of a cardiac problem between participants with an income below the poverty threshold compared to those equal to or above it.

Associations were found between suffering heart failure (*p* < 0.001), angina pectoris (*p* < 0.05), heart attack (*p* < 0.001), and stroke (*p* < 0.001) and having an income below the poverty threshold (Figure 4). Individuals with a family income below the poverty line had a significantly higher prevalence of cardiac pathologies: congestive heart failure, angina pectoris, heart attack, and stroke. Likewise, the Chi-squared test found no association between suffering coronary heart disease and having an income below the poverty threshold, and the difference in the prevalence of this cardiac pathology between participants with an income below the threshold and those with a higher income was not significant according to the z-test.

Significant associations were found between having an income below the poverty line and suffering two or more (<0.001), three or more (<0.001), four or more (<0.001), and five or more comorbidities (<0.001). However, no associations were found between this adverse economic situation and suffering no comorbidity or suffering one or more comorbidities (Table 2).

**Figure 5:**
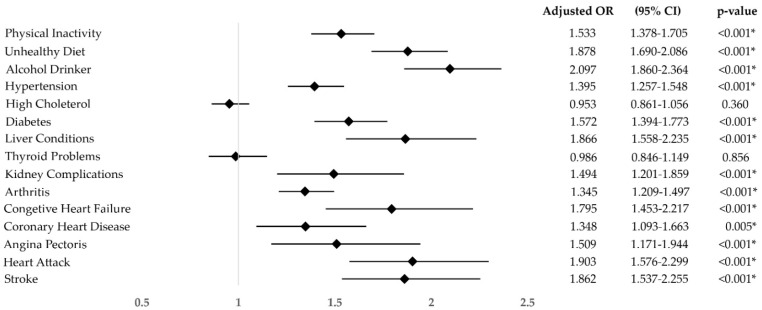
Odds ratio of suffering non-communicable diseases and of non-healthy habits, adjusted for age and sex, of middle-aged and older adults with a family income below the poverty threshold compared to those with a higher income. * = *p*-value under the level of significance.

The odds ratio analysis revealed that middle-aged and older adults with a family income below the poverty threshold had increased odds of being physically inactive (OR: 1.533; 95%CI: 1.378–1.705), having an unhealthy diet (OR: 1.878; 95%CI: 1.690–2.086), being or have been an alcohol drinker (OR: 2.097; 95%CI: 1.860–2.364), suffering hypertension (OR: 1.395; 95%CI: 1.257–1.548), diabetes (OR: 1.572; 95%CI: 1.394–1.773), liver conditions (OR: 1.866; 95%CI: 1.558–2.235), kidney complications (OR: 1.494; 95%CI: 1.201–1.859), arthritis (OR: 1.345; 95%CI: 1.209–1.497), congestive heart failure (OR: 1.795; 95%CI: 1.453–2.217), coronary heart disease (OR: 1.348; 95%CI: 1.093–1.663), angina pectoris (OR: 1.509; 95%CI: 1.171–1.944), heart attack (OR: 1.903; 95%CI: 1.576–2.299), and stroke (OR: 1.862; 95%CI: 1.537–2.255), compared to those with a higher income (Figure 5). In contrast, the odds ratios of suffering high cholesterol (*p* = 0.360) and thyroid problems (*p* = 0.856) of people with such adverse economic situation were not significant.

**Figure 6:**
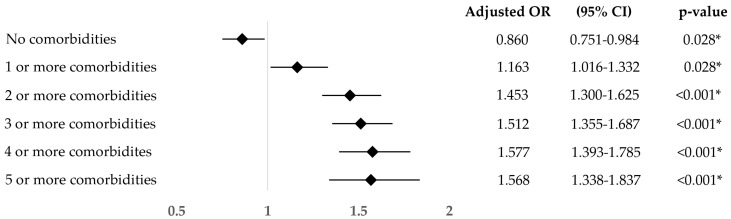
Odds ratio, adjusted for age and sex, of suffering comorbidities of middle-aged and older adults with an income below the poverty threshold compared to those with a higher income. * = *p*-value under the level of significance.

The odds ratio of suffering comorbidities evidenced that people with an income below the poverty line had reduced odds of not suffering any comorbidity (OR: 0.860; 95%CI: 0.751–0.984), likewise they had significantly higher odds than people with better economic situation of suffering one or more (OR: 1. 163; 95%CI: 1.016–1.332), two or more (OR: 1.453; 95%CI: 1.300–1.625), three or more (OR: 1.512; 95%CI: 1.355–1.785), four or more (OR: 1.577; 95%CI: 1.393–1.785), and five or more comorbidities (OR: 1.568; 95%CI: 1.338–1.837) (Figure 6).

## 4. Discussion

This study aimed to explore if there are associations between having an income below the poverty threshold and having unhealthy lifestyle habits, suffering from non-communicable diseases, and suffering multimorbidity. The results of this research confirmed that in middle-aged and older US adults, there exist associations between a family income below the poverty threshold and physical inactivity, eating an unhealthy diet, and being or having been an alcoholic. Similarly, associations were found between this adverse economic situation and suffering from hypertension, diabetes, liver condition, kidney problems, arthritis, angina pectoris, heart attack, coronary heart disease, and stroke. Furthermore, relationships between this adverse economic situation and high cholesterol, thyroid problems, and coronary heart disease, were not found. Finally, a family income below the poverty line was associated with suffering two or more, three or more, four or more, and five or more comorbidities.

The results showed that there were associations between a family income below the poverty line, and physical inactivity, an unhealthy diet, and being or having been a high alcohol drinker. These non-healthy lifestyle habits were shown to be more prevalent in individuals in this adverse economic situation, in this line, increased odds ratio of having these behaviors were seen for the group with a family income considered as poverty. Regarding physical activity, Chien et al. observed a significant positive association between the prevalence of physical inactivity and the percentage of poverty, that is, people who were economically disadvantaged had a higher incidence of physical inactivity, coinciding with our results [27]. Household income level has also been shown to influence the healthfulness of food intake, so that lower incomes were associated with poorer food quality as well as being characterized by a diet considered unhealthy [28].

Regarding alcohol consumption, a previous study found a significant positive association between drinking alcoholic beverages and poverty level, reporting that drinking alcoholic beverages increases the probability of belonging to a poor household, in line with our study [29]. In addition, it has been reported that the rate of alcoholism increases as socioeconomic status decreases, placing drinkers and ex-drinkers, mostly, in a lower socioeconomic class [30], reaffirming our findings.

Considering the influence of having a low socioeconomic status on the likelihood of having an unhealthy lifestyle, it can be affirmed that socioeconomic status affects the exposure to health risk factors [31,32]. Thus, there is a need to consider the health consequences that this may have for the population in this disadvantaged economic situation. Recent studies indicate that increased salt intake, fatty foods, physical inactivity, and alcohol consumption are common risk factors linked to hypertension [33]. However, there are other risk factors related to socioeconomic disadvantage, such as early childhood undernutrition, lower educational level, place of residence, and employment status, which increase the likelihood of non-communicable diseases like hypertension and cardiovascular diseases [12,34,35].

Associations between having an income under the poverty threshold and suffering hypertension, diabetes, liver condition, kidney problems, arthritis, coronary heart disease, angina pectoris, heart attack, and stroke were confirmed. At the same time a significantly higher prevalence of these non-communicable diseases was found in those with this economically disadvantaged situation. The analysis of the odds ratio adjusted for gender and age corroborated the increased likelihood of suffering these health problems for the population under the poverty threshold. In line with this, associations have been found that connect economic level and educational level with the prevalence of pathologies such as hypertension, especially with increasing age [36]. Other research has found that people with incomes below the poverty line suffer from a higher incidence of diabetes [37,38] and high cholesterol [39]. Additionally, it has been identified that people in a disadvantaged economic situation suffer to a greater extent from liver [40], thyroid [41], kidney [42], and arthritis [43] problems.

Socioeconomic status (SES) has been inversely related to cardiovascular disease incidence, as shown in the present study, and also with mortality [44]. In developed countries, individuals with a low SES present higher odds of heart disease and cardiovascular disease in comparison to those with a higher SES, with marked differences in the incidence of these health problems between these population groups [45,46]. Thus, by highlighting the inverse relationship between socioeconomic status (SES) and the risk factors described above, it is necessary to establish preventive measures to reduce the impact of non-communicable diseases in the population with a low SES [47], justifying the approach of the present study.

Finally, the study of the number of coexisting comorbidities revealed that there were associations between having two or more, three or more, four or more, and five or more comorbidities an having a family income below the poverty line. Finding that the percentage of the population suffering from two, three, four, and five or more comorbidities is significantly higher in the low-income population compared to those with an income equal to or above the poverty threshold. The analysis of the odds ratio evidenced that people with this adverse economic situation were significantly more likely to have one or more, two or more, three or more, four or more, and five or more comorbidities, while they have a lower chance of not having any comorbidities. Evidence suggests that economically disadvantaged people suffer from a higher number comorbidities, linking this to the deprivation and social inequalities experienced by this part of the population [48,49,50]. On the contrary, a higher SES has been shown to be associated with a greater life expectancy, improved subjective well-being, and a superior medical history than people with a lower SES [51], which is in line with the findings of the present research highlighting that there are difference in the incidence of comorbidities according to SES.

The main relevance of our study is to expose the main health risks faced by the economically disadvantaged and to make visible through our findings that the prevalence of having certain non-communicable pathologies could be greatly reduced by focusing attention on modifiable risk factors, i.e., by placing lifestyle habits at a key point in the health of the most disadvantaged people. This study offers a novel and original contribution by comprehensively exploring the associations between poverty, unhealthy habits, comorbidity, and non-communicable diseases in middle-aged and older adults in the US. The originality lies in the holistic approach, the identification of a range of interrelated diseases and health behaviors, and the clear implications for public policy action, with an emphasis on improving the health and well-being of economically disadvantaged populations.

### 4.1. Practical Implications and Future Line Research

The findings of the present study cand help public health institutions and administrations to promote programs that seek to increased adherence to healthy behaviors in terms of improving diet quality, increasing physical activity, and reducing or eliminating alcohol intake in economically disadvantaged populations. Focusing resources on modifiable risk factors would imply a considerable reduction in health expenditure for public institutions and for individuals who are part of this group with a higher risk of suffering from health problems, which is the population with low incomes. Furthermore, this research and other similar works also aim to increase the visibility of the health implications that the population in economic disadvantaged situations face, and if possible, to help people in this situation to become aware of the importance of lifestyle in terms of the state of their health.

In this sense, the results of this study could serve as a reference for future experimental studies in low-income population groups, including intervention programs on re-education and adherence to healthy behaviors, as well as to explore the effect of these programs in longitudinal studies.

### 4.2. Limitations

This study has some limitations that should be acknowledged. First, due to its cross-sectional design, causality cannot be established between household income below the poverty line and the unhealthy habits, non-communicable diseases, and multimorbidities observed. Second, the data regarding lifestyle habits were derived from self-reported responses in the NHANES, which may be subject to recall bias and social desirability bias. Finally, this study analyzed multiple health outcomes independently using binary logistic regression, which increases the risk of Type I errors due to multiple comparisons. Therefore, the results should be interpreted with caution, considering both statistical significance and theoretical relevance. Future longitudinal research is needed to further explore these relationships and study causality among the studied variables.

## 5. Conclusions

The present research showed that, in a population of middle-aged and older US adults, there are associations among having a family income below the poverty line and physical inactivity, an unhealthy diet, high alcohol consumption, having two or more comorbidities, and suffering from the following non-communicable diseases: hypertension, diabetes, liver problems, kidney problems, arthritis, heart failure, angina pectoris, heart attack, and stroke. Additionally, significantly higher prevalences of these non-healthy habits, diseases, and multimorbidity were found for middle-aged and older adults in this economically disadvantaged situation. Increased odds ratios of having these unhealthy habits and non-communicable diseases, plus coronary heart disease, were found for this group. Also, higher odds of having one or more comorbidities and reduced chances of not experiencing any comorbidity were observed for individuals under the poverty line. This evidence could be used as a benchmark for public health institutions to allocate greater resources to programs for adherence to healthy behaviors in populations with family incomes below the poverty line, avoiding a situation of social exclusion and discrimination. Identifying the main risks to which economically disadvantaged families are exposed could lead to increased awareness and involvement in the reduction in modifiable risk factors related to unhealthy lifestyle habits.

## Figures and Tables

**Table 1:** Characterization of the sample, middle-aged and older adults from the NHANES 2011–2020.

Variable	General Population (n = 10,788)		Males (n = 5653)		Females (n = 5135)		*p* *
Age							
Mean (SD)	59.40 (11.87)		59.80 (11.86)		58.97 (11.87)		-
Median (IQR)	59.00 (40)		60.00 (40)		58.00 (40)		<0.001
Family income	General Population (n = 10,788)		Males (n = 5653)		Females (n = 5135)		*p*
	n	%	n	%	n	%	
Equal/over poverty threshold	8919	82.7%	4687	82.4%	4232	82.9%	0.496
Under poverty threshold	1869	17.3%	966	17.1%	903	17.6%	

*p* * = *p*-value of the Mann–Whitney U test; *p* = *p*-value of the Chi-squared test; * = *p*-value under the level of significance.

**Table 2:** Prevalence of multimorbidity in middle-aged and older adults with a family income under the poverty threshold, compared to those with a higher income, and associations of multimorbidity with having a family income under the poverty threshold.

		Equal/Above Poverty Threshold		Under Poverty Threshold		X^2^	Phi	*p*-Value
		N	(%)	N	(%)			
No morbidities	Yes	1760	19.7	345	18.5	1.597	0.012	0.206
	No	7159	80.3	1524	81.5			
1 or more comorbidities	Yes	7159	80.3	1524	81.5	1.597	−0.012	0.206
	No	1760	19.7	345	18.5			
2 or more comorbidities	Yes	5125	57.5	1184 *	63.3	22.061	−0.045	<0.001
	No	3794	42.5	685 *	36.7			
3 or more comorbidities	Yes	3297	37.0	811 *	43.4	27.062	−0.050	<0.001
	No	5622	63.0	1058 *	56.6			
4 or more comorbidities	Yes	1809	20.3	479 *	25.6	26.428	−0.049	<0.001
	No	7110	79.7	1390 *	74.4			
5 or more comorbidities	Yes	887	9.9	243 *	13.0	15.394	−0.038	<0.001
	No	8032	90.1	1626 *	87.0			

* = significant differences in the z-test with Bonferroni corrections between participants with an income below the poverty threshold compared to those equal to or above it.

## Data Availability

The data used in this research are publicly available and can be accessed at the following link: https://www.cdc.gov/nchs/nhanes/index.htm (accessed on 13 May 2024).

## References

[1] Vera-Romero O.E., Vera-Romero F.M. (2013). Evaluación del nivel socioeconómico: Presentación de una escala adaptada en una población de Lambayeque. Rev. Cuerpo Méd. Hosp. Nac. Almanzor Aguinaga Asenjo.

[2] Bigé N., Hejblum G., Baudel J.-L., Carron A., Chevalier S., Pichereau C., Maury E., Guidet B. (2015). Homeless Patients in the ICU: An Observational Propensity-Matched Cohort Study. Crit. Care Med..

[3] Lyon S.M., Benson N.M., Cooke C.R., Iwashyna T.J., Ratcliffe S.J., Kahn J.M. (2011). The Effect of Insurance Status on Mortality and Procedural Use in Critically Ill Patients. Am. J. Respir. Crit. Care Med..

[4] Rush B., Wiskar K., Celi L.A., Walley K.R., Russell J.A., McDermid R.C., Boyd J.H. (2018). Association of Household Income Level and in-Hospital Mortality in Patients with Sepsis: A Nationwide Retrospective Cohort Analysis. J. Intensive Care Med..

[5] Chandola T., Conibere R. (2015). Social Exclusion, Social Deprivation and Health. International Encyclopedia of the Social & Behavioral Sciences.

[6] Benaïs M., Duprey M., Federici L., Arnaout M., Mora P., Amouretti M., Bourgeon-Ghittori I., Gaudry S., Garçon P., Reuter D. (2024). Association of Socioeconomic Deprivation with Outcomes in Critically Ill Adult Patients: An Observational Prospective Multicenter Cohort Study. Ann. Intensive Care.

[7] World Health Organization (2020). Sources of Care in Mixed Health Systems. https://www.who.int/data/gho/data/themes/topics/indicator-groups/sources-of-care-in-mixed-health-systems.

[8] Claxton G., Rae M., Panchal N., Whitmore H., Damico A., Kenward K., Long M. (2015). Health Benefits in 2015: Stable Trends in the Employer Market. Health Aff..

[9] Tikkanen R., Abrams M.K. (2020). Health Care from a Global Perspective, 2019: Higher Spending, Worse Outcomes? The Commonwealth Fund. Issue Briefs. https://www.commonwealthfund.org/publications/issue-briefs/2020/jan/us-health-care-global-perspective-2019.

[10] Sortsø C., Lauridsen J., Emneus M., Green A., Jensen P.B. (2017). Socioeconomic Inequality of Diabetes Patients’ Health Care Utilization in Denmark. Health Econ. Rev..

[11] Cox A.M., McKevitt C., Rudd A.G., Wolfe C.D. (2006). Socioeconomic Status and Stroke. Lancet Neurol..

[12] Gheorghe A., Griffiths U., Murphy A., Legido-Quigley H., Lamptey P., Perel P. (2018). The Economic Burden of Cardiovascular Disease and Hypertension in Low- and Middle-Income Countries: A Systematic Review. BMC Public Health.

[13] Yusuf S., Joseph P., Rangarajan S., Islam S., Mente A., Hystad P., Brauer M., Kutty V.R., Gupta R., Wielgosz A. (2020). Modifiable Risk Factors, Cardiovascular Disease, and Mortality in 155 722 Individuals from 21 High-Income, Middle-Income, and Low-Income Countries (PURE): A Prospective Cohort Study. Lancet.

[14] Hsu Y.-C., Cheng S.Y.-H., Chien M.-N., Cheng S.-P. (2023). Impact of Social and Economic Factors on Global Thyroid Cancer Incidence and Mortality. Eur. Arch. Oto-Rhino-Laryngol..

[15] Le M.H., Devaki P., Ha N.B., Jun D.W., Te H.S., Cheung R.C., Nguyen M.H. (2017). Prevalence of Non-Alcoholic Fatty Liver Disease and Risk Factors for Advanced Fibrosis and Mortality in the United States. PLoS ONE.

[16] Pinto-Meza A., Moneta M.V., Alonso J., Angermeyer M.C., Bruffaerts R., Caldas De Almeida J.M., De Girolamo G., De Graaf R., Florescu S., Kovess Masfety V. (2013). Social Inequalities in Mental Health: Results from the EU Contribution to the World Mental Health Surveys Initiative. Soc. Psychiatry Psychiatr. Epidemiol..

[17] Asogwa O.A., Boateng D., Marzà-Florensa A., Peters S., Levitt N., Van Olmen J., Klipstein-Grobusch K. (2022). Multimorbidity of Non-Communicable Diseases in Low-Income and Middle-Income Countries: A Systematic Review and Meta-Analysis. BMJ Open.

[18] Mendenhall E., Kohrt B.A., Norris S.A., Ndetei D., Prabhakaran D. (2017). Non-Communicable Disease Syndemics: Poverty, Depression, and Diabetes among Low-Income Populations. Lancet.

[19] Wood W., Quinn J.M., Kashy D.A. (2002). Habits in Everyday Life: Thought, Emotion, and Action. J. Personal. Soc. Psychol..

[20] Rippe J.M. (2018). Lifestyle Medicine: The Health Promoting Power of Daily Habits and Practices. Am. J. Lifestyle Med..

[21] Salvy S.-J., Miles J.N.V., Shih R.A., Tucker J.S., D’Amico E.J. (2017). Neighborhood, Family and Peer-Level Predictors of Obesity-Related Health Behaviors Among Young Adolescents. J. Pediatr. Psychol..

[22] Chokshi D.A. (2018). Income, Poverty, and Health Inequality. JAMA.

[23] Cunningham C., O’ Sullivan R., Caserotti P., Tully M.A. (2020). Consequences of Physical Inactivity in Older Adults: A Systematic Review of Reviews and Meta-analyses. Scand. J. Med. Sci. Sports.

[24] Jayedi A., Soltani S., Abdolshahi A., Shab-Bidar S. (2020). Healthy and Unhealthy Dietary Patterns and the Risk of Chronic Disease: An Umbrella Review of Meta-Analyses of Prospective Cohort Studies. Br. J. Nutr..

[25] Sohi I., Franklin A., Chrystoja B., Wettlaufer A., Rehm J., Shield K. (2021). The Global Impact of Alcohol Consumption on Premature Mortality and Health in 2016. Nutrients.

[26] Bull F.C., Maslin T.S., Armstrong T. (2009). Global Physical Activity Questionnaire (GPAQ): Nine Country Reliability and Validity Study. J. Phys. Act. Health.

[27] Chien L.C., Li X., Staudt A. (2017). Physical Inactivity Displays a Mediator Role in the Association of Diabetes and Poverty: A Spatiotemporal Analysis. Geospat. Health.

[28] Ssewanyana D., Abubakar A., Van Baar A., Mwangala P.N., Newton C.R. (2018). Perspectives on Underlying Factors for Unhealthy Diet and Sedentary Lifestyle of Adolescents at a Kenyan Coastal Setting. Front. Public Health.

[29] Jayathilaka R., Selvanathan S., Bandaralage J.S. (2016). Is There a Link between Alcohol Consumption and the Level of Poverty?. Appl. Econ..

[30] Fitzgerald H.E., Zucker R.A. (2019). Socioeconomic Status and Alcoholism: The Contextual Structure of Developmental Pathways to Addiction. Children of Poverty.

[31] Langenberg C., Kuh D., Wadsworth M.E.J., Brunner E., Hardy R. (2006). Social Circumstances and Education: Life Course Origins of Social Inequalities in Metabolic Risk in a Prospective National Birth Cohort. Am. J. Public Health.

[32] Brunner E., Shipley M.J., Blane D., Smith G.D., Marmot M.G. (1999). When Does Cardiovascular Risk Start? Past and Present Socioeconomic Circumstances and Risk Factors in Adulthood. J. Epidemiol. Community Health.

[33] Chowdhury M.A.B., Uddin M.J., Haque M.R., Ibrahimou B. (2016). Hypertension among Adults in Bangladesh: Evidence from a National Cross-Sectional Survey. BMC Cardiovasc. Disord..

[34] Deaton C., Froelicher E.S., Wu L.H., Ho C., Shishani K., Jaarsma T. (2011). The Global Burden of Cardiovascular Disease. Eur. J. Cardiovasc. Nurs..

[35] Nduka C., Uthman O., Kimani P., Stranges S. (2015). Drug Abuse in People Living with HIV in the Era of Highly Active Antiretroviral Therapy: A Systematic Review and Meta-Analysis. J. Addict. Res. Ther..

[36] Abba M.S., Nduka C.U., Anjorin S., Mohamed S.F., Agogo E., Uthman O.A. (2021). Influence of Contextual Socioeconomic Position on Hypertension Risk in Low- and Middle-Income Countries: Disentangling Context from Composition. BMC Public Health.

[37] Buescher P.A., Whitmire J.T., Pullen-Smith B. (2010). Medical Care Costs for Diabetes Associated with Health Disparities among Adult Medicaid Enrollees in North Carolina. N. C. Med. J..

[38] Chapel J.M., Ritchey M.D., Zhang D., Wang G. (2017). Prevalence and Medical Costs of Chronic Diseases Among Adult Medicaid Beneficiaries. Am. J. Prev. Med..

[39] Mosca I., Bhuachalla B.N., Kenny R.A. (2013). Explaining Significant Differences in Subjective and Objective Measures of Cardiovascular Health: Evidence for the Socioeconomic Gradient in a Population-Based Study. BMC Cardiovasc. Disord..

[40] Major J.M., Sargent J.D., Graubard B.I., Carlos H.A., Hollenbeck A.R., Altekruse S.F., Freedman N.D., McGlynn K.A. (2014). Local Geographic Variation in Chronic Liver Disease and Hepatocellular Carcinoma: Contributions of Socioeconomic Deprivation, Alcohol Retail Outlets, and Lifestyle. Ann. Epidemiol..

[41] Almubarak A.A., Albkiry Y.A., Alsalem A.A., Elkrim Saad M.A. (2021). The Association of Low Socioeconomic Status with Advanced Stage Thyroid Cancer. J. Taibah Univ. Med. Sci..

[42] Begaj I., Khosla S., Ray D., Sharif A. (2013). Socioeconomic Deprivation Is Independently Associated with Mortality Post Kidney Transplantation. Kidney Int..

[43] Brennan-Olsen S.L., Taillieu T.L., Turner S., Bolton J., Quirk S.E., Gomez F., Duckham R.L., Hosking S.M., Duque G., Green D. (2019). Arthritis in Adults, Socioeconomic Factors, and the Moderating Role of Childhood Maltreatment: Cross-Sectional Data from the National Epidemiological Survey on Alcohol and Related Conditions. Osteoporos. Int..

[44] De Mestral C., Stringhini S. (2017). Socioeconomic Status and Cardiovascular Disease: An Update. Curr. Cardiol. Rep..

[45] Lopez A.D., Adair T. (2019). Is the Long-Term Decline in Cardiovascular-Disease Mortality in High-Income Countries over? Evidence from National Vital Statistics. Int. J. Epidemiol..

[46] Davari M., Maracy M.R., Khorasani E. (2019). Socioeconomic Status, Cardiac Risk Factors, and Cardiovascular Disease: A Novel Approach to Determination of This Association. ARYA Atheroscler..

[47] Stewart J., Manmathan G., Wilkinson P. (2017). Primary Prevention of Cardiovascular Disease: A Review of Contemporary Guidance and Literature. JRSM Ardiovascular Dis..

[48] Callan M.J., Kim H., Matthews W.J. (2015). Predicting Self-Rated Mental and Physical Health: The Contributions of Subjective Socioeconomic Status and Personal Relative Deprivation. Front. Psychol..

[49] Kraus M.W., Adler N., Chen T.-W.D. (2013). Is the Association of Subjective SES and Self-Rated Health Confounded by Negative Mood? An Experimental Approach. Health Psychol..

[50] Mishra S., Carleton R.N. (2015). Subjective Relative Deprivation Is Associated with Poorer Physical and Mental Health. Soc. Sci. Med..

[51] Adler N.E., Rehkopf D.H. (2008). U.S. Disparities in Health: Descriptions, Causes, and Mechanisms. Annu. Rev. Public Health.

